# Concordance of SARS-CoV-2 RNA in Aerosols From a Nurses Station and in Nurses and Patients During a Hospital Ward Outbreak

**DOI:** 10.1001/jamanetworkopen.2022.16176

**Published:** 2022-06-08

**Authors:** Rebecca A. Stern, Michael E. Charness, Kalpana Gupta, Petros Koutrakis, Katherine Linsenmeyer, Rebecca Madjarov, Marco A. G. Martins, Bernardo Lemos, Scot E. Dowd, Eric Garshick

**Affiliations:** 1 Department of Environmental Health, Harvard T.H. Chan School of Public Heath, Boston, Massachusetts; 2 Veterans Affairs Boston Healthcare System, West Roxbury, Boston, Massachusetts; 3 Harvard Medical School, Boston, Massachusetts; 4 Boston University School of Medicine, Boston, Massachusetts; 5 Department of Neurology, Brigham and Women’s Hospital, Boston, Massachusetts; 6 Department of Environmental Health and Molecular and Integrative Physiological Sciences Program, Harvard T.H. Chan School of Public Health, Boston, Massachusetts; 7 Molecular Research LP (MR DNA), Shallowater, Texas; 8 Pulmonary, Allergy, Sleep, and Critical Care Medicine Section, Veterans Affairs Boston Healthcare System, West Roxbury, Boston, Massachusetts; 9 Channing Division of Network Medicine, Brigham and Women’s Hospital, Boston, Massachusetts

## Abstract

**Importance:**

Aerosol-borne SARS-CoV-2 has not been linked specifically to nosocomial outbreaks.

**Objective:**

To explore the genomic concordance of SARS-CoV-2 from aerosol particles of various sizes and infected nurses and patients during a nosocomial outbreak of COVID-19.

**Design, Setting, and Participants:**

This cohort study included patients and nursing staff in a US Department of Veterans Affairs inpatient hospital unit and long-term-care facility during a COVID-19 outbreak between December 27, 2020, and January 8, 2021. Outbreak contact tracing was conducted using exposure histories and screening with reverse transcriptase–polymerase chain reaction (RT-PCR) for SARS-CoV-2. Size-selective particle samplers were deployed in diverse clinical areas of a multicampus health care system from November 2020 to March 2021. Viral genomic sequences from infected nurses and patients were sequenced and compared with ward nurses station aerosol samples.

**Exposure:**

SARS-CoV-2.

**Main Outcomes and Measures:**

The primary outcome was positive RT-PCR results and genomic similarity between SARS-CoV-2 RNA in aerosols and human samples. Air samplers were used to detect SARS-CoV-2 RNA in aerosols on hospital units where health care personnel were or were not under routine surveillance for SARS-CoV-2 infection.

**Results:**

A total of 510 size-fractionated air particle samples were collected. Samples representing 3 size fractions (>10 μm, 2.5-10 μm, and <2.5 μm) obtained at the nurses station were positive for SARS-CoV-2 during the outbreak (3 of 30 samples [10%]) and negative during 9 other collection periods. SARS-CoV-2 partial genome sequences for the smallest particle fraction were 100% identical with all 3 human samples; the remaining size fractions shared >99.9% sequence identity with the human samples. Fragments of SARS-CoV-2 RNA were detected by RT-PCR in 24 of 300 samples (8.0%) in units where health care personnel were not under surveillance and 7 of 210 samples (3.3%; *P* = .03) where they were under surveillance.

**Conclusions and Relevance:**

In this cohort study, the finding of genetically identical SARS-CoV-2 RNA fragments in aerosols obtained from a nurses station and in human samples during a nosocomial outbreak suggests that aerosols may have contributed to hospital transmission. Surveillance, along with ventilation, masking, and distancing, may reduce the introduction of community-acquired SARS-CoV-2 into aerosols on hospital wards, thereby reducing the risk of hospital transmission.

## Key Points

**Question:**

Is SARS-CoV-2 RNA found in aerosols in hospital break rooms and nurses stations during a nosocomial outbreak?

**Findings:**

In this cohort study, SARS-CoV-2 genome sequences in air samples collected at a nurses station were identified in all particle sizes and were identical to human samples from a nosocomial outbreak. Detection of aerosol-borne SARS-CoV-2 was statistically less frequent on units under surveillance (7 of 210 samples) than without surveillance (24 of 300 samples).

**Meaning:**

These findings suggest that nosocomial infection may result from aerosol-borne SARS-CoV-2 introduced by employees and patients into common hospital areas; surveillance may help reduce the introduction of SARS-CoV-2 into aerosols.

## Teaser

This cohort study uses air samplers placed in hospital ward nurses stations and break rooms to detect aerosol-borne SARS-CoV-2 RNA during a nosocomial outbreak.

## Introduction

Increasing evidence indicates that COVID-19 may be transmitted through aerosols. Aerosols smaller than 2.5 μm can remain airborne for several hours, travel beyond 6 ft, and transport SARS-CoV-2 into the lower respiratory tract.^1^ Not surprisingly, SARS-CoV-2 RNA has been identified in air samples from rooms or wards housing unmasked patients with COVID-19.^2,3,4,5,6^ A previous study revealed that SARS-COV-2 may be identified within a range of aerosol sizes from hospital settings remote from the direct care of patients with COVID-19.^7^ Positive samples were collected most frequently in hospital areas where health care personnel (HCP) congregate and where masking may be less consistent, such as nurses stations.^7^ The frequency of positive samples was associated with the community prevalence of SARS-CoV-2 infection, consistent with the introduction of community-acquired SARS-COV-2 into the hospital setting.^7,8,9^

These findings led to the hypothesis that nosocomial transmission of COVID-19 might be associated with the aerosolization of community-acquired SARS-CoV-2 within hospital spaces shared by HCP and patients. To test this hypothesis, we began to sample air in multiple shared hospital spaces before a looming surge in COVID-19 cases in the fall of 2020. Our goal was to improve infection prevention strategies through better understanding of locations with positive air samples and nosocomial SARS-CoV-2 infections.

## Methods

This cohort study followed the Strengthening the Reporting of Observational Studies in Epidemiology (STROBE) reporting guideline. A waiver was granted by the Veterans Affairs Boston Healthcare System (VABHS) Institutional Review Board because the data were gathered in the setting of a quality improvement project. The Veterans Affairs Boston Research and Development Committee approved the sampling procedures.

### Air Sampling Analysis

We conducted size-selective surveillance for airborne SARS-CoV-2 at 2 campuses of the VABHS between November 16, 2020, and March 11, 2021, using a microenvironmental cascade impactor that collects airborne particles in 3 size ranges: larger than 10.0 μm, 2.5 to 10.0 μm, and smaller than 2.5 μm.^7^ Samples were collected approximately every week, with a break from December 10, 2020, to January 4, 2021 (eTable 1 in the Supplement).

The cascade inlets were located at breathing-zone height. Ten 72-hour sampling sessions were conducted in the 154-bed acute, tertiary care hospital at the West Roxbury campus and an 80-bed subacute and long-term residential facility at the Brockton campus. Airborne particles were analyzed for viral RNA using reverse transcriptase–polymerase chain reaction (RT-PCR) targeting the N1 and N1/N2 genes, as previously described^7^ and detailed in the eMethods in the Supplement. Selected samples underwent genomic shotgun sequencing. Samples with cycle threshold (Ct) values below 40 on the Centers for Disease Control and Prevention RT-PCR assay were sent for shotgun sequencing; choice of which additional samples were sequenced is detailed in the eMethods in the Supplement. Mean temperature and relative humidity during the sampling periods were 23.2 °C and 18.1% for West Roxbury and 23.8 °C and 20.0% for Brockton (eTable 2 in the Supplement). During this time, HCP at the Brockton facility and ward B at the West Roxbury facility were under biweekly to twice-weekly surveillance for SARS-CoV-2 infection using RT-PCR and BinaxNOW antigen testing (Abbott Laboratories), as previously described.^9^

The ventilation system for the outbreak ward (ward A) consisted of recirculated air through fan coil units with fresh air intake. Air exchange was estimated at 6 to 8 air changes per hour, 2 of which were from outside air. There were ceiling grates at both ends of the ward and over the middle of the nurses station.

### Outbreak Investigation

We investigated an outbreak among 103 HCP and patients that occurred between December 27, 2020, and January 8, 2021, on a non–COVID-19 medical ward (ward A) at West Roxbury. A case was defined as a person with a positive RT-PCR result for SARS-CoV-2 between January 2 and 8 and no history of COVID-19 infection in the previous 90 days. Contact tracing was conducted using exposure histories and RT-PCR testing of nasopharyngeal samples or antigen testing (BinaxNOW) of midnasal turbinate samples.^9^ Antigen testing was conducted 2 to 3 times per week on all contacts until there were no further positive results. All positive antigen tests were confirmed by RT-PCR, and selected SARS-CoV-2 samples were sequenced at the Massachusetts Department of Public Health or The Jackson Laboratory.

### Statistical Analysis

Differences in proportions were analyzed using the χ^2^ test, and statistical significance was defined as 2-sided *P* < .05. Python, version 2.7 software^10^ and the package Scipy^11^ were used for data analysis.

## Results

### Outbreak

This study was a clinical disease outbreak investigation; therefore, no demographic data were systematically collected. The outbreak on ward A was first detected when a nurse (N1) became symptomatic 4 days after a first-dose administration of the mRNA-1273 vaccine (Moderna) and tested positive for SARS-CoV-2 (Figure, A). This individual was presumed to be the index case based on the chronology of symptoms and testing. Contact tracing over the next 6 days based on RT-PCR and antigen test results identified SARS-CoV-2 infection in an additional 8 nurses and 8 patients from ward A and 2 nurses from ward B. A total of 34 nursing staff from ward A and 50 other close contacts of nursing staff and patients tested negative during the 8-day follow-up period.

**Figure.**
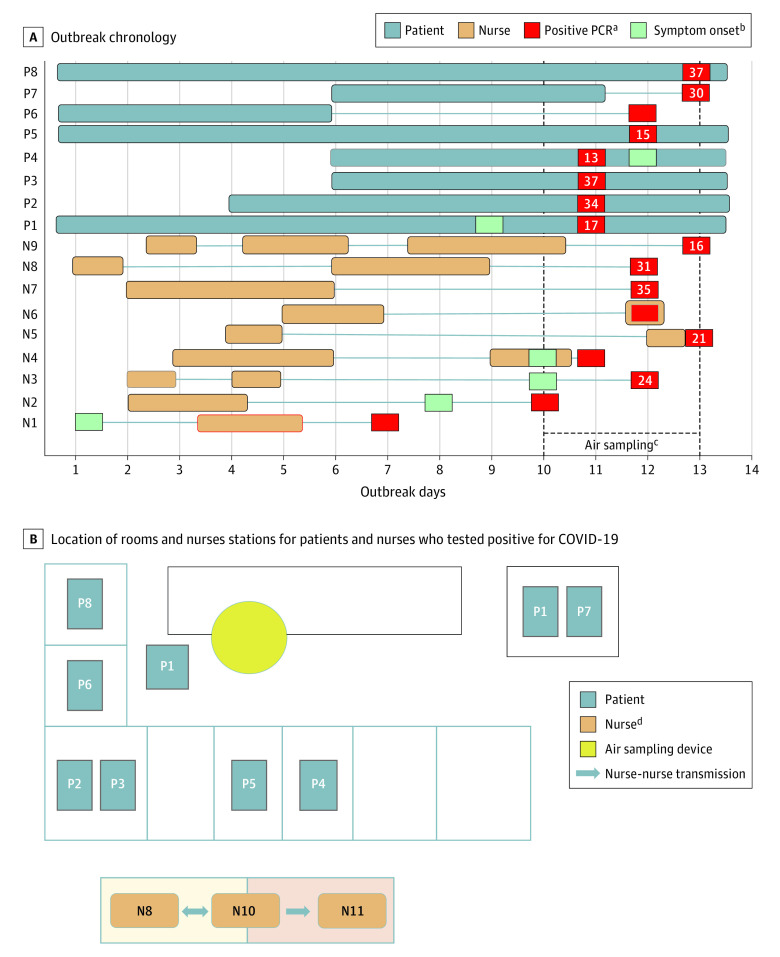
COVID-19 Outbreak Timeline and Ward A Layout The timeline shows outbreak days 1 to 13, with nurse 1 (N1) presumed to be the outbreak source on the basis of earliest symptom onset. The patient room entrances were located 9 to 19 ft from the nurses station. Patient 1 (P1) wandered and spent time in front of the nurses station. The remaining patients occupied single- or double-occupancy rooms. ^a^Reverse transcriptase–polymerase chain reaction (PCR) cycle threshold (Ct) values shown; empty boxes indicate no available Ct value. ^b^No green box indicates that a patient or nurse was asymptomatic during the observation period. ^c^Collector at the nurses station was sampling air between study days 10 and 13. ^d^N10 and N11 were from a different ward; N10 tested positive on outbreak day 6 with a Ct of 15, and N11 tested positive on outbreak day 10 with a Ct of 17. N8 and N10 had a high-risk exposure to each other in the community before N10 tested positive.

The infected nurses on ward A worked shifts on nonconsecutive days (Figure, A). Although they were not all present together throughout the outbreak, all 9 infected nurses on ward A had exposure to at least 1 other nurse or patient within the cluster. All 8 infected patients were potentially exposed to an infected nurse or another infected patient on ward A. Patient rooms on this ward were not under negative pressure, and when patients were diagnosed with COVID-19, they were transferred to a COVID-19 unit. Nurses on this ward wore surgical masks. Patients were unmasked inside their rooms and wore surgical masks outside their rooms.

Four infected nurses and 7 infected patients were on ward A during 3 days of coincidental air sampling at the ward A nurses station, including 3 nurses and 3 patients with Ct values less than 24. Patient 1 (Ct = 17) often wandered or sat unmasked in front of the nurses station approximately 10 to 15 feet from the sampler, and nurses at the nurses station would occasionally lower their masks to drink. The remaining infected patients were confined to their rooms, except when undergoing testing in other hospital locations.

There was insufficient information to determine the direction of transmission among most nurses and patients. Nurse 10 from ward B had a high-risk community exposure with nurse 8 from ward A and became symptomatic 4 days before nurse 11 on ward B (Figure, B). Therefore, nurse 10 was the presumed source for transmission from ward A to ward B. Indeed, viral genome sequences from nurse 11 shared 99.99% to 100% identity with those from nurse 3 and patient 4 from ward A (Table 1), consistent with a common source of infection.

**Table 1.** Sequence Homology of Air and Human Samples During a COVID-19 Outbreak

Location	Genome coverage of sample, %^b^	Particle size, μm	Identity with ward A human samples, %^a^		
			Human 1	Human 2	Human 3
**Air samples** ^c^ ^,^ ^d^					
Ward C break room 2	16	<2.5	98.90	98.90	98.90
	19	2.5-10	99.36	99.36	99.36
	12	>10	94.38	94.38	94.38
Ward C nurses station (negative pressure)	23	<2.5	99.80	99.80	99.80
	17	2.5-10	98.51	98.51	98.51
	26	>10	99.86	99.86	99.86
Ward A nurses station	14	<2.5	100.0	100.0	100.0
	59	2.5-10	99.91	99.91	99.91
	24	>10	99.97	99.97	99.97
**Human samples (ward A)**					
Human 1	NA	NA	NA	100.0	99.99
Human 2	NA	NA	100.0	NA	100.0
Human 3	NA	NA	99.99	100.0	NA

Abbreviation: NA, not applicable.

^a^ One sequence (ward A, 2.5-10 μm) was accepted into GenBank under accession No. OL304239. Sequences for human samples 1, 2, and 3 were submitted to GenBank under accession Nos. MW540299, MW540300, and MW540301, respectively.

^b^ Percentage of genome (RNA) isolated compared against GenBank accession No. MW540301 (human 3).

^c^ Ward C (COVID-19 unit) had negative pressure ventilation during this sampling period, including the nurses station. Ward C break room and ward A nurses station had usual hospital ventilation.

^d^ Within-sampler homology stages in the ward C break room for particle sizes 2.5 to 10 and larger than 10 μm were 100% identical; at the ward C nurses station, all stages were different. At the ward A nurses station, all stages were 100% identical. Samplers operated concurrently on the different wards.

The outbreak began within 2 weeks of the first availability of mRNA vaccines; hence, among 11 infected nursing staff, 5 received a first shot of mRNA-1273 vaccine less than 14 days before the outbreak, and 5 were unvaccinated. Vaccination status was unknown for 1 staff member. Because our vaccination effort targeted HCP first, none of the patients were vaccinated at the time of the outbreak.

### Air Sampling on Outbreak Ward

Samples representing 3 size fractions (<2.5 μm, 2.5-10 μm, and >10 μm) obtained at the nurses station were positive for SARS-CoV-2 (3 of 30 samples [10%]) during the outbreak and negative during 9 other weekly collection periods. Fragments of SARS-CoV-2 RNA in the smallest aerosols (<2.5 μm) in ward A showed 100% sequence identity with the human samples (Table 1). The other size fractions in ward A had greater homology with the human samples (2.5-10 μm, 99.91%; >10 μm, 99.97%) than did samples collected over the same dates on ward C, a COVID-19 unit (nurses station: <2.5 μm, 99.80%; 2.5-10 μm, 98.51%; >10 μm, 99.86%; break room 2: <2.5 μm, 98.90%; 2.5-10 μm, 99.36%; >10 μm, 94.38%).

### Air Sampling Across the Medical Center

Ongoing surveillance of HCP on selected units of the medical center provided an opportunity to determine whether active surveillance and isolation of infected HCP were associated with a reduced prevalence of SARS-CoV-2 RNA in air samples from those units. Fragments of SARS-CoV-2 RNA were detected by RT-PCR in 24 of 300 samples (8.0%) in units across the medical center where HCP were not under surveillance and 7 of 210 (3.3%) in units where HCP were under surveillance (*P* = .03) (Table 2, Table 3). Approximately one-half (20 of 38 [52.6%]) of all positive samples came from the 2.5- to 10-μm size fraction, with the remainder split between the smaller than 2.5 μm (7 [18.4%]) and larger than 10 μm (11 [28.9%]) size fractions. Thirty of 37 air samples (81%) were positive for SARS-CoV-2 genomic RNA by shotgun sequencing (eTable 3 in the Supplement). Six of 7 samples (86%) that were negative by RT-PCR were positive by sequencing. Fragment sequences ranged from 6% to 60% of the SARS-CoV-2 genome, and none aligned with other human coronaviruses.

**Table 2.** Positive Samples Based on Reverse Transcriptase–Polymerase Chain Reaction by Location and Aerosol Particle Size

Location^a^	Particle size			Samples	
	<2.5 μm	2.5-10 μm	>10 μm	Total No.	Positive, No. (%)
HCP not under surveillance					
WR					
Negative pressure locations	1	3	3	69	7 (10.1)
Nurses stations	1	2	2	93	5 (5.4)
Transit or leisure areas^b^	1	2	2	48	5 (10.4)
Staff break rooms	2	5	0	120	7 (5.8)
Total	5	12	7	300	24 (8.0)^c^
HCP under surveillance					
BR and WR provider workroom and nurses stations	0	2	0	90	2 (2.2)
BR transit or leisure areas^d^	1	0	1	60	2 (3.3)
BR patient or staff break rooms	0	2	1	60	3 (5.0)
Total	1	4	2	210	7 (3.3)^c^

Abbreviations: BR, Veterans Affairs Boston Healthcare System, Brockton; HCP, health care personnel; WR, Veterans Affairs Boston Healthcare System, West Roxbury.

^a^ Table 3 lists the specific locations included in each location category.

^b^ Family waiting room.

^c^ *P* = .03 by χ^2^ test.

^d^ Patient day room; transit area is the hallway or similar area.

**Table 3.** Positive Samples Based on Reverse Transcriptase–Polymerase Chain Reaction by Detailed Location and Aerosol Particle Size

Location	Particle size			Samples	
	<2.5 μm	2.5-10 μm	>10 μm	Total No.	Positive, No. (%)
HCP not under surveillance					
WR negative pressure locations					
Ward C^a^ nurses station	0	2	0	18	2 (11.1)
Ward C corridor	1	1	3	30	5 (16.7)
MICU					
Nurses station	0	0	0	9	0
PPE doffing area (11 × 22 ft)	0	0	0	12	0
Total	1	3	3	69	7 (10.1)
WR nurses stations					
Outside ward C	0	0	0	12	0
MICU	0	1	1	21	2 (9.5)
Ward A	1	1	1	30	3 (10.0)
Total	1	2	2	63	5 (7.9)
WR transit/leisure areas^b^					
Hallway outside main ICU entrance	0	2	1	30	3 (10.0)
MICU					
Family waiting area (14 × 15 ft)	0	0	0	9	0
Exit room (11 × 22 ft)	1	0	1	9	2 (22.2)
Total	1	2	2	48	5 (10.4)
WR staff break rooms					
Ward C 1 (10 × 13 ft)	0	0	0	12	0
Ward C 2 (14 × 15 ft)	1	4	0	18	5 (27.8)
MICU 1 (11 × 22 ft)	0	1	0	9	1 (11.1)
MICU 2 (14 × 15 ft)	0	0	0	21	0
Ward A (14 × 17 ft)	1	0	0	30	1 (3.3)
Ward B (13 × 17 ft)	0	0	0	30	0
Total	2	5	0	120	7 (5.8)
WR total	5	12	7	300	24 (8.0)
HCP under surveillance					
BR (subacute/long-term care) and WR provider workroom and nurses station					
WR ward B	0	0	0	30	0
Subacute medical ward provider workroom (9 × 15 ft)	0	2	0	30	2 (6.7)
Long-term-care ward nurses station	0	0	0	30	0
Total	0	2	0	90	2 (2.2)
BR transit/leisure areas^c^					
Building main lobby	1	0	0	30	1 (3.3)
Long-term-care ward patient day room (14 × 25 ft)	0	0	1	30	1 (3.3)
Total	1	0	1	60	2 (3.3)
BR patient/staff break rooms					
Subacute medical ward patient/staff dining area (24 × 40 ft)	0	0	0	6	0
Subacute medical ward staff break room (16 × 18 ft)	0	2	1	24	3 (12.5)
Long-term-care ward patient/staff dining area (24 × 40 ft)	0	0	0	30	0
Total	0	2	1	60	3 (5.0)
HCP under surveillance total	2	8	4	210	7 (3.3)

Abbreviations: BR, Veterans Affairs Boston Healthcare System, Brockton; HCP, health care personnel; ICU, intensive care unit; MICU, medical intensive care unit; PPE, personal protective equipment; WR, Veterans Affairs Boston Healthcare System, West Roxbury.

^a^ Ward C is the COVID-19 unit.

^b^ Family waiting room.

^c^ Patient day room; transit area is the hallway or similar area.

## Discussion

In this cohort study, nosocomial transmission of SARS-CoV-2 occurred on a medical unit during coincidental collection of air samples, and several observations were consistent with aerosol transmission. The temporal sequence of transmission suggested that the infection was introduced by a symptomatic nurse and spread among nurses and patients. At least 6 nurses and patients who were present during air sample collection had nasopharyngeal samples with a Ct less than 25, a range associated with shedding of replication-competent virus,^12^ and most were early in their illness, when detection of viral RNA in exhaled aerosols is most frequent.^13^ Viral sequences from 3 infected persons were nearly identical, suggesting nosocomial transmission from a common source. Finally, SARS-CoV-2 RNA genomic fragments in the smallest aerosols collected at the nurses station shared sequence identity with the human samples. Respiratory viruses tend to concentrate most in these smallest aerosols.^14^

The origin of SARS-CoV-2 RNA in aerosol samples at the nurses station on ward A is unclear. As many as 4 infected nurses worked at the nurses station within a few feet of the sample collector, and 1 or more of these nurses may have introduced SARS-CoV-2 into airborne particles collected at that location. A second possible source was the patient who spent time in front of the nurses station near the sample collector. A less likely source was the remaining cohort of infected patients on ward A because they were confined to their rooms at a greater distance from the sample collector.

The presence of SARS-CoV-2 in aerosols at the nurses station on ward A was temporally associated with the outbreak; in contrast, SARS-CoV-2 RNA was not detected at the same location during 9 other weeks. This temporal and spatial association along with the genetic similarity of the aerosol and human samples establishes a potential link between the human and air samples. The data do not directly implicate those aerosol samples in the etiology of this outbreak or establish the direction of transmission.

Broad sampling across the VABHS during a COVID-19 wave revealed SARS-CoV-2 RNA in aerosols from multiple hospital locations remote from the care of patients with COVID-19. Air samples obtained on units where HCP were subject to routine surveillance had a significantly lower positivity rate than air samples from units where HCP surveillance was not conducted. These differences were not likely to be due to differences in community prevalence^7^ because most units under surveillance were in Brockton, where community prevalence was consistently higher than in West Roxbury.^15^ These findings suggest that surveillance, in conjunction with interventions including distancing, masking, and ventilation, may reduce the introduction of community-acquired SARS-CoV-2 into aerosols on hospital wards, consistent with the observation that surveillance is associated with a reduction in nosocomial transmission of SARS-CoV-2.^9^ With current technology, air sampling surveillance does not have sufficiently rapid turnaround time to monitor nosocomial infection in real time; however, these sampling data were useful in suggesting the presumed mode of transmission.

### Limitations

This study had several limitations. The lack of sampling between December 10, 2020, and January 4, 2021, likely missed a substantial number of positive samples because this period was at the height of the COVID-19 incidence curve in Massachusetts.^15^ The RT-PCR test may have underestimated the frequency of positive samples because shotgun sequencing of several Centers for Disease Control and Prevention assay-negative samples also revealed SARS-CoV-2 genomic fragments. Although RT-PCR may be less sensitive than shotgun sequencing, it was valuable in helping to identify hospital areas with the highest frequency of aerosol samples positive for SARS-CoV-2. Sequencing and Ct values were not available for all infected HCP and patients, limiting our ability to track transmission. Likewise, the collection of air samples for 72 hours inevitably leads to RNA degradation, reducing the availability of large genomic fragments for comparison with human samples and precluding precise quantitation for comparison with the infectious dose. Although sequences from available aerosol RNA fragments were nearly identical with human samples, there might have been differences in uncovered segments of the aerosol viral genome. Finally, the fractionated air collection method precluded viral culture, so we were unable to determine whether aerosol samples contained replication-competent virus.

## Conclusions

This cohort study found a presence of genetically concordant SARS-CoV-2 RNA fragments in various-sized aerosols obtained from a nurses station and in human samples during a nosocomial outbreak, suggesting that aerosol transmission across long and short distances may have contributed to hospital transmission. Surveillance and isolation of infected HCP may reduce the introduction of community-acquired SARS-CoV-2 into aerosols on hospital wards, thereby potentially reducing the risk of hospital transmission. Improvements in air filtration, ventilation, and masking in shared hospital spaces may further decrease transmission of SARS-CoV-2 and other airborne respiratory viruses.

## Notes

Supplement. **eMethods.** Detailed Methods **eTable 1.** Sampling Dates **eTable 2.** Temperature and Relative Humidity (RH) During Sampling Periods **eTable 3.** Positive Samples Using Shotgun Sequencing
