# Error in Data

**DOI:** 10.1001/jamanetworkopen.2022.21256

**Published:** 2022-06-30

In the Original Investigation titled “Concordance of SARS-CoV-2 RNA in Aerosols From a Nurses Station and in Nurses and Patients During a Hospital Ward Outbreak,”^1^ published June 8, 2022, a row of data regarding surveillance in the WR Ward B staff break room had been incorrectly placed within Table 2, which affected some related values in the table and text (the correct number of samples in units under surveillance is 240; the correct number of samples in units without surveillance is 270). The number of health care personnel and patients involved in the outbreak was also clarified in the Outbreak Investigation section of the Methods (there were 19 total infections, not 103), and The Funding/Support section was updated to include missing funding from the National Institute of Environmental Health Sciences to Dr Stern. This article has been corrected.^1^
