# Supplementary material for: Concordance of SARS-CoV-2 RNA in Aerosols From a Nurses Station and in Nurses and Patients During a Hospital Ward Outbreak
Source: JAMA Netw Open. 2022 Jun 8;5(6):e2216176. doi: 10.1001/jamanetworkopen.2022.16176 (PMC9178433; doi:10.1001/jamanetworkopen.2022.16176)
Supplement: Supplement. — eMethods. Detailed Methods eTable 1. Sampling Dates eTable 2. Temperature and Relative Humidity (RH) During Sampling Periods eTable 3. Positive Samples Using Shotgun Sequencing [file jamanetwopen-e2216176-s001.pdf]

Supplemental Online Content

Stern RA, Charness ME, Gupta K, et al. Concordance of SARS-CoV-2 RNA in aerosols from a nurses station and in nurses and patients during a hospital ward outbreak. *JAMA Netw Open*. 2022;5(6):e2216176. 10.1001/jamanetworkopen.2022.16176

eMethods. Detailed Methods

eTable 1. Sampling Dates

eTable 2. Temperature and Relative Humidity (RH) During Sampling Periods

eTable 3. Positive Samples Using Shotgun Sequencing

This supplemental material has been provided by the authors to give readers additional information about their work.

© 2022 Stern RA et al, *JAMA Network Open.*

eMethods. Detailed Methods

Sampling

Samplers were placed 45 to 58 inches above the floor at West Roxbury (WR) and 38 to 59 inches above the floor at Brockton (BR). Field blanks were placed in hospital areas but not exposed to air flow. Samples and blanks were processed simultaneously. Mean temperature and relative humidity for WR and BR during sampling periods were 23.2ºC and 18.1% and 23.8ºC and 20.0%, respectively (eTable 2).

RT-PCR Methods:

2019-nCoV CDC qPCR probe assays

CDC assay procedures were repeated following Stern et al. (2021).[7] Viral RNA was extracted from the samples using the RNeasy Mini Kit (Qiagen, Hilden, Germany) following the manufacturer's instructions. Samples were first vortexed for 1 minute and 400-500 µl sample with RNA later was used for RNA extraction. 5.6 µg Poly-A carrier RNA (Qiagen, Hilden, Germany) was also mixed with each sample for extraction. Carrier RNA enhances the low copy viral nucleic acids binding to the mini column and reduces the chance of viral RNA degradation. RNA was eluted in 40 µl RNase free water. RNA quantity and quality were determined using NanoDrop2000 (Thermo Scientific, Waltham, MA, USA). Samples were then used to quantify the viral concentrations by qPCR using 2019-nCoV CDC qPCR probe assays (Integrated DNA Technologies, Inc., Coralville, IA, USA). 12 ul of RNA sample was first used for cDNA synthesis using QuantiTect Reverse Transcription kit (Qiagen, Hilden, Germany). 2 µl of the synthesized cDNA was used to perform the qPCR reaction using 2X PrimeTime Gene Expression Master Mix (Integrated DNA Technologies, Inc., Coralville, IA, USA) in StepOnePlus Real-Time PCR System (Applied Biosystems). The qPCR reaction was carried out with an initial holding stage of 95˚C for 3 minutes for PCR enzyme activation. The cycling stage consisted of 50 cycles of 95˚C for 5 seconds, followed by 55˚C for 30 seconds. Genomic RNA from Severe Acute Respiratory Syndrome Coronavirus 2 (2019-nCoV/USA-WA1/2020; ATCC, Manassas, VA, USA) was used as standard.

N1/SYBR Green PCR Assay

All samples were also subjected to an N1 gene assay, which contrasts with the CDC method that uses the N1 and N2 regions together. 2 µl of the synthesized cDNA was used to perform the enrichment PCR for 35 cycles using

© 2022 Stern RA et al, *JAMA Network Open.*

SARSCOV2 primers (SARSCOV2_F25, R24, F26, R26). The PCR reaction was carried out with an initial holding stage of 95˚C for 5 minutes for PCR enzyme activation. The cycling stage consisted of 35 cycles of 94˚C for 30 seconds, followed by 53˚C for 30 seconds, 72˚C for 120 seconds and final extension 72˚C for 10 minutes. PCR product (25 µl) was cleaned using 1X PCRClean DX (Aline Biosciences) beads and eluted in 25 ul AE buffer. 2 µl of the cleaned amplicon or 2µl of unenriched cDNA was used to perform the qPCR reaction using 2X SYBR Green qPCR Master Mix (Applied Biosystems) in QuantStudio Real-Time PCR System (Applied Biosystems). The qPCR reaction was carried out with an initial holding stage of 50˚C for 2 minutes followed by 95˚C for 10 minutes for PCR enzyme activation. The cycling stage consisted of 45 cycles of 95˚C for 30 seconds, 54˚C for 40 seconds followed by 72˚C for 60 seconds. After the PCR run, a melting curve analysis was performed to assess the qPCR products. Genomic RNA from Severe acute respiratory syndrome-related coronavirus 2 (2019-nCoV/USA-WA1/2020; ATCC) was used as standard.

© 2022 Stern RA et al, *JAMA Network Open.*

Identification of samples for submitting to shotgun sequencing

We used a multi-pronged approach to identify samples that would be submitted for sequencing.

First, we used RT-qPCR using the Centers for Disease Control & Prevention (CDC) assay based on the N1/N2 gene as well as the N1/SYBR Green PCR assay. Positive samples were identified as those with a cycle threshold (C_t) cutoff of ≤40 based on the CDC Assay. All samples positive based on the CDC Assay (30) were submitted for sequencing. Next, we identified putative positives using the SYBR Green approach, and samples with a melting point of 80°C indicated a potential positive sample. One sample was positive only on the SYBR Green but not on the CDC assay and was included in the shotgun sequencing. One sample that was not positive on the CDC or SYBR Green assays was also included. Finally, the other stages (five in total) from the same cascade of four positive samples based on the CDC assay were included in the shotgun sequencing submission. In total, this multi-tiered approach allowed us to select 37 samples for shotgun sequencing.

Shotgun sequencing

One sequences with the greatest genome coverage, about 60% of the genome, was accepted into GenBank (Accession #OL304239). Samples from shotgun sequencing were deemed positive if they had sections of the genome that matched the SARS-CoV-2 genome based on NCBI Blast and negative if they returned only primer matches.

© 2022 Stern RA et al, *JAMA Network Open.*

eTable 1. Sampling dates

**Brockton**	**West Roxbury**
11/16/20-11/19/20	11/17/20-11/20/20
11/30/20-12/03/20	12/01/20-12/04/20
12/07/20-12/10/20	12/08/20-12/11/20
01/04/21-01/07/21	01/05/20-01/08/20
1/11/21-1/14/21	01/12/21-01/15/21
1/18/21-1/21/21	01/19/21-01/22/21
1/25/21-1/28/21	01/26/21-01/29/21
2/08/21-2/11/21	02/09/21-02/12/21
2/22/21-2/25/21	02/23/21-02/26/21
3/08/21-3/11/21	03/09/21-03/12/21

© 2022 Stern RA et al, *JAMA Network Open.*

eTable 2. Temperature and relative humidity (RH) during sampling periods

	**West Roxbury Campus**		**Brockton Campus**	
	Temp. (ºC)	RH (%)	Temp. (ºC)	RH (%)
Mean	23.2	18.1	23.8	20.0
Max	25.5	27.5	27.3	34.2
Min	20.6	15.0	21.6	15.0
St. Dev.	1.0	3.0	1.3	4.6

© 2022 Stern RA et al, *JAMA Network Open.*

eTable 3. Positive samples using shotgun sequencing

**Location**	**Total Positive**	**Total Shotgun Samples**	**Positive Sampling Period Dates (December 2020 to March 2021)**
**West Roxbury**			
**Locations Under Negative Pressure**			
Ward C[a] Nurses Station	4	4	1/5, 1/12
Ward C Corridor	3	4	12/1, 1/12, 3/9
MICU Nurses Station	N/A	N/A	
MICU PPE Doffing Area	N/A	N/A	
**Nurses Stations**			
Ward C Nurses station	N/A	N/A	
MICU Nurses Station	3	3	12/8, 1/26, 3/9
Ward B Nurses Station	N/A	N/A	
Ward A Nurses Station	4	4	12/8, 1/5
**Transit/Leisure Areas**			
Hallway Outside Main ICU Entrance	3	3	11/17, 3/9
MICU Family Waiting Area	N/A	N/A	
MICU Exit Room	1	2	2/9
**Staff Breakrooms**			
Ward C Breakroom 1	N/A	N/A	
Ward C Breakroom 2	7	7	12/8, 1/5, 1/12, 1/26, 2/9
MICU Breakroom 1	1	1	12/8
MICU Breakroom 2	N/A	N/A	
Ward B Breakroom	N/A	N/A	
Ward A Breakroom	1	1	1/19
**Brockton**			
**Provider Workroom and Nurses Station**			
Subacute Medical Ward Provider Workroom	2	3	11/16, 12/7
Long-term Care Ward Nurses Station	N/A	N/A	

© 2022 Stern RA et al, *JAMA Network Open.*

**Transit/Leisure Areas**			
Building Main Lobby	0	1	
Long-term Care Ward Patient Day Room	0	1	
**Patient/Staff Breakrooms**			
Subacute Medical Ward Patient/Staff Dining Area	N/A	N/A	
Subacute Medical Ward Staff Breakroom	1	3	1/25
Long-term Care Ward Patient/Staff Dining Area	N/A	N/A	

[a]Ward C is the COVID patient care ward.

© 2022 Stern RA et al, *JAMA Network Open.*
